# Effects of generative artificial intelligence on cognitive effort and task performance: study protocol for a randomized controlled experiment among college students

**DOI:** 10.1186/s13063-025-08950-3

**Published:** 2025-07-11

**Authors:** Youjie Chen, Yingying Wang, Torsten Wüstenberg, Rene F. Kizilcec, Yiwen Fan, Yanfei Li, Bin Lu, Meng Yuan, Junlai Zhang, Ziyue Zhang, Pascal Geldsetzer, Simiao Chen, Till Bärnighausen

**Affiliations:** 1 Department of Information Science, Cornell University, Ithaca, USA; 2 Heidelberg Institute of Global Health (HIGH), Faculty of Medicine and University Hospital, Heidelberg University, Heidelberg, Germany; 3 Neuroimaging for Language, Literacy and Learning Laboratory, Department of Special Education and Communication Disorders, University of Nebraska-Lincoln, Lincoln, NE USA; 4 Core Facility for Neuroscience of Self-Regulation (CNSR), Heidelberg University, Field of Focus 4 (FoF4), Heidelberg, Germany; 5 Center for Clinical and Epidemiologic Research, Institute of Heart, Lung and Blood Vessel Diseases, Beijing Anzhen Hospital, Capital Medical University, Beijing , Beijing, China; 6 Department of Cancer Epidemiology, National Cancer Center/National Clinical Research Center for Cancer/Cancer Hospital, Chinese Academy of Medical Sciences and Peking Union Medical College, Beijing, China; 7 Peking Union Medical College, Beijing, China; 8 Department of Economics, Vienna University of Economics and Business (WU), Vienna, Austria; 9 Institute of Public Health and Nursing Research (IPP), Faculty 11 Health and Human Sciences, Bremen University, Bremen, Germany; 10 Department of Medicine, Stanford University School of Medicine, Stanford, USA; 11 Chinese Academy of Medical Sciences and Peking Union Medical College, Beijing, China

**Keywords:** Generative artificial intelligence, Randomized controlled trial, Human cognition, Cognitive effort, Critical thinking, Analytical writing, Eye-tracking, Functional near-infrared spectroscopy

## Abstract

**Background:**

The advancement of generative artificial intelligence (AI) has shown great potential to enhance productivity in many cognitive tasks. However, concerns are raised that the use of generative AI may erode human cognition due to over-reliance. Conversely, others argue that generative AI holds the promise to augment human cognition by automating menial tasks and offering insights that extend one’s cognitive abilities. To better understand the role of generative AI in human cognition, we study how college students use a generative AI tool to support their analytical writing in an educational context. We will examine the effect of using generative AI on cognitive effort, a major aspect of human cognition that reflects the extent of mental resources an individual allocates during the cognitive process. We will also examine the effect on writing performance achieved through the human-generative AI collaboration.

**Methods:**

This study is a randomized controlled lab experiment that compares the effects of using generative AI (intervention group) versus not using it (control group) on cognitive effort and writing performance in an analytical writing task designed as a hypothetical writing class assignment for college students. During the experiment, eye-tracking technology will monitor eye movements and pupil dilation. Functional near-infrared spectroscopy (fNIRS) will collect brain hemodynamic responses. A survey will measure individuals’ perceptions of the writing task and their attitudes on generative AI. We will recruit 160 participants (aged 18–35 years) from a German university where the research will be conducted.

**Discussion:**

This trial aims to establish the causal effects of generative AI on cognitive effort and task performance through a randomized controlled experiment. The findings aim to offer insights for policymakers in regulating generative AI and inform the responsible design and use of generative AI tools.

Trial registration.

ClinicalTrials.gov NCT06511102. Registered on July 15, 2024. https://clinicaltrials.gov/study/NCT06511102

## Administrative information

Note: the numbers in curly brackets in this protocol refer to SPIRIT checklist item numbers. The order of the items has been modified to group similar items (see http://www.equator-network.org/reporting-guidelines/spirit-2013-statement-defining-standard-protocol-items-for-clinical-trials/).

**Table:** 

Title {1}	Effects of generative artificial intelligence on cognitive effort and task performance: study protocol for a randomized controlled experiment among college students
Trial registration {2a and 2b}	ClinicalTrials.gov NCT06511102. Registered on July 15, 2024.
Protocol version {3}	V1.0, Sep 2024
Funding {4}	This study is funded by Horizon Europe (HORIZON-MSCA-2021-SE-01) (Project 101,086,139—PoPMeD-SuSDeV), the Chinese Academy of Medical Sciences and Peking Union Medical College (Project 2024-CFT-QT-034), and Alexander von Humboldt-Stiftung Award.
Author details {5a}	Youjie Chen: Department of Information Science, Cornell University, USA; Heidelberg Institute of Global Health (HIGH), Faculty of Medicine and University Hospital, Heidelberg University, Germany Yingying Wang: Neuroimaging for Language, Literacy and Learning Laboratory, Department of Special Education and Communication Disorders, University of Nebraska-Lincoln, Lincoln, NE, USA Torsten Wüstenberg: Core Facility for Neuroscience of Self-Regulation (CNSR), Field of Focus 4 (FoF4), Heidelberg University, Germany Rene F. Kizilcec: Department of Information Science, Cornell University, USA Yiwen Fan: Heidelberg Institute of Global Health (HIGH), Faculty of Medicine and University Hospital, Heidelberg University, Germany Yanfei Li: Heidelberg Institute of Global Health (HIGH), Faculty of Medicine and University Hospital, Heidelberg University, Germany Bin Lu: Center for Clinical and Epidemiologic Research, Beijing Anzhen Hospital, Capital Medical University, Beijing Institute of Heart, Lung and Blood Vessel Diseases, Beijing, China; Department of Cancer Epidemiology, National Cancer Center/National Clinical Research Center for Cancer/Cancer Hospital, Chinese Academy of Medical Sciences and Peking Union Medical College, Beijing, China Meng Yuan: Peking Union Medical College, China Junlai Zhang: Department of Economics, Vienna University of Economics and Business (WU), Vienna, Austria; Heidelberg Institute of Global Health (HIGH), Faculty of Medicine and University Hospital, Heidelberg University, Heidelberg, Germany Ziyue Zhang: Institute of Public Health and Nursing Research (IPP), Faculty 11 Health and Human Sciences, Bremen University, Germany; Heidelberg Institute of Global Health (HIGH), Faculty of Medicine and University Hospital, Heidelberg University, Germany Pascal Geldsetzer: Department of Medicine, Stanford University School of Medicine, Stanford, USA Simiao Chen: Heidelberg Institute of Global Health (HIGH), Faculty of Medicine and University Hospital, Heidelberg University, Germany; Chinese Academy of Medical Sciences and Peking Union Medical College, China Till Bärnighausen: Heidelberg Institute of Global Health (HIGH), Faculty of Medicine and University Hospital, Heidelberg University, Germany
Name and contact information for the trial sponsor {5b}	European Commission. https://commission.europa.eu/about/contact_en
Role of sponsor {5c}	The funders will have no role in the study design; collection, management, analysis, and interpretation of data; writing of the report; or submission decisions.

## Introduction

### Background and rationale {6a}

Recent advances in generative artificial intelligence (AI) have raised heated debates regarding its use in performing cognitive tasks. Human collaboration with generative AI tools, such as OpenAI’s ChatGPT, has been shown to enhance productivity across a wide range of cognitive tasks, including professional writing tasks among white-collar workers [1], customer support services [2], knowledge-intensive consulting [3], creative story-writing [4], and creative ideation [5]. However, concerns have been raised that heavy use of these tools may lead to the erosion of human cognition [6, 7], which has important implications for human cognitive health [8].

Many prior technological innovations have raised similar concerns about potentially causing a deleterious effect on human cognition and cognitive health. For example, the use of calculators may hinder arithmetic literacy, the use of search engines may reduce aspects of memory skills [9], and the use of social media may contribute to everyday cognitive lapses [10]. According to these concerns, access to these tools may allow individuals to bypass effortful tasks and thus reduce opportunities to engage in the mental practice required for cognitive abilities to fully develop in the human brain [11, 12]. However, technologies could also be seen as an extension of human cognition, or the so-called "extended mind" [13]. With appropriate cognitive offloading, technologies can extend the limits of human cognition and become an active component of human brain mechanisms [14, 15]. For example, the use of calculators can help individuals circumvent tedious arithmetic calculations and focus on complex mathematical problems. The use of search engines can stimulate learning by broadening individuals’ knowledge space and providing tools for self-regulated learning [16]. In the end, the effect of technology tools on human cognition is a nuanced problem that depends on the cognitive task, the tool itself, and how it is used.

The emergence of generative AI tools has again raised heated debates about the effects of the new technology on human cognition due to its significant advancements over its antecedents [17]. These advancements include the following: First, unlike traditional tools that assist with basic skills, such as calculators, generative AI exhibits a higher level of intelligence to create ideas and construct arguments. Second, generative AI encompasses a broad range of cognitive skills rather than a well-defined single one. Consequently, it is difficult to pinpoint which cognitive skills generative AI may affect. Third, generative AI is continuously developing at an ever-increasing speed, which complicates our ability to predict the kinds of cognitive skills it may affect in the future. In consideration of all these advancements and their implications, it is important to evaluate the effects of generative AI tool use on human cognition.

Recent studies have begun to shed light on how generative AI may affect human cognition, mainly through its effects on learning performance outcomes [18]. Randomized controlled trials have found that students performed better when using general-purpose generative AI tools but performed worse when these tools were taken away [19, 20]. This suggests that students may have relied on the tool to bypass cognitive processes essential for developing cognitive skills, which ultimately compromised their performance. A subsequent study has found that generative AI boosted learning for those who use it to engage in deep conversations and explanations but hampered learning for those who sought direct answers [21]. This finding further highlights the difference between using generative AI as an active extension of human cognition and using it merely for passive cognitive offloading.

Studies so far have gained preliminary insights into generative AI’s effects on human cognition and performance through a standard assessment paradigm (SAP) [22]. In their experiments, participants were randomly assigned to either have or not have access to generative AI, and their skills were then tested through task performance in isolation from AI support [19–21]. However, this approach only captures a static snapshot of the *learning product* but is insufficient to understand the ongoing developmental *learning process* during human-generative AI interaction [23]. To gain a deeper understanding of generative AI’s effects on human cognition, it is important to develop measures during the human-generative AI interaction process. In contrast to learning products, the learning process can reveal authentic learning progress over time [24]. Additionally, the SAP tends to focus on performance outcomes but may overlook activities for long-term cognitive development that can be better evaluated through process-based behavioral measures [25, 26].

In light of this background, our study will evaluate the effects of generative AI on task performance and cognitive effort during the human-generative AI interaction process. The task performance will reflect the overall achievement of an individual executing a specified cognitive task in collaboration with generative AI. Cognitive effort will reflect the extent to which the individual actively utilizes their mental resources while performing the task. Exerting cognitive effort is fundamental to training one’s cognitive abilities and maintaining the fitness of the human brain [11]. Measuring cognitive effort during generative AI use allows us to assess whether the technology primarily reduces individuals’ mental exertion or facilitates individuals to invest more effort. We will use state-of-the-art technology to evaluate psychophysiological proxies of cognitive effort throughout the task process in a lab-based randomized controlled trial (RCT). Specifically, we will use an eye tracker to measure pupil dilation changes and a functional near-infrared spectroscopy (fNIRS) to measure cortical hemodynamic activity. Our study context will focus on analytical writing among college students. We choose analytical writing because this task requires high cognitive effort to develop critical thinking [27, 28], a fundamental higher-order thinking skill crucial for problem-solving and decision-making [29, 30]. As important as critical thinking skill is, it remains empirically unclear whether the use of generative AI has implications for the development of this skill.

### Objectives {7}

Our study aims to achieve the following objectives:

1. To establish the effects of generative AI on human cognition and task performance, measured by cognitive effort during the writing process and analytical writing performance (primary objective).
2. To explore the effects of generative AI on subjective perceptions of health and learning-related outcomes.
3. To investigate heterogeneous treatment effects across individuals with different characteristics, such as their motivation to perform well in the writing task, English language ability, writing ability, and critical thinking ability.

### Trial design {8}

**Fig. 1:**
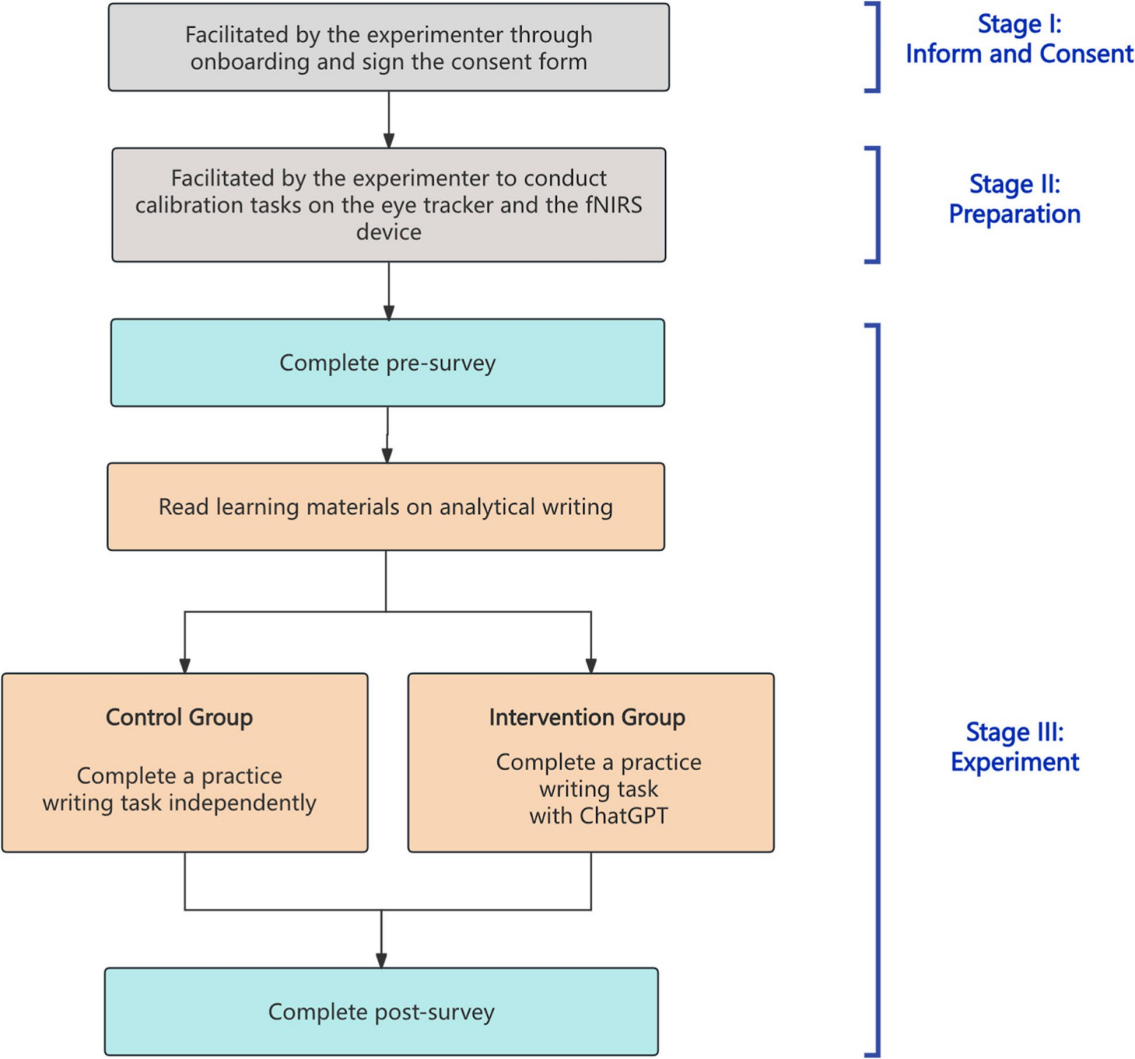
The trial design with participants randomized into the intervention group and the control group in a 1:1 ratio

Our study is a parallel RCT that compares the effects of using ChatGPT (intervention group) versus not using ChatGPT (control group) in an analytical writing task (Fig. 1). Participants will be randomized in a 1:1 ratio. The study follows an exploratory framework.

The experiment will be conducted on one participant at a time. For each participant, the study consists of three stages. In the first stage, the experimenter will onboard the participant and ask the participant to sign a consent form. In the second stage, the participant will be invited into an experiment room to sit in front of a computer with eye-tracking functionality that collects data on eye movements and pupil size. An experimenter will assist the participant in wearing an fNIRS device that collects data on brain hemodynamic responses. In the third stage, the actual experiment will begin. The participant will independently follow the instructions displayed on the computer screen. The instructions will frame the entire experiment as a writing class that the participant has taken, together with all other participants in the study. To mimic the context in a typical educational setting, the instruction will explain that the participant’s writing score will be graded and ranked among others in the class. The participant will receive their writing score and the class average after the study is completed among all participants.

Within the third stage, the participant will first complete a pre-survey. They will then be instructed to read some materials to learn about analytical writing, and then practice what they have learned by writing an analytical essay as their homework assignment. During practice, the instructions will outline the writing prompt, writing requirement, time requirement, grading feedback, and grading rubric. Specifically, the participant will be asked to write a 350–600-word essay stating whether they agree or disagree with the writing prompt. The time requirement will explain that there is no time limit, but participants are recommended to spend 30 min on the task. We intentionally designed the writing task without a time limit to better reflect a typical homework setting, in contrast to an exam setting where time is constrained. This distinction may elicit different writing behaviors and patterns of using AI tools. For grading feedback, the instructions will explain that the participant will receive their writing score, the class average, and writing feedback from expert instructors once the entire experiment ends. Participants in the intervention group can use ChatGPT to support their writing. Specifically, we choose GPT-4 because it has been independently benchmarked for its standalone performance on analytical writing in the Graduate Record Examinations (GRE), as documented in OpenAI’s technical report [31]. This provides a reference point for us to evaluate the analytical writing capabilities during human-generative AI collaboration. Participants in the control group will complete the writing task without AI assistance. After the writing task, the participant will complete a post-survey. The entire study will last for approximately 1.5 h for each participant.

## Methods: participants, interventions, and outcomes

### Study setting {9}

This study will be a lab experiment conducted at Heidelberg University in Germany. Participants are college students who will be recruited through social media platforms, email lists, and flyers. During the preparation stage, an experimenter will guide the participant into an experiment room and instruct them to sit in front of a computer. For the main part of the experiment, the participant will independently follow instructions displayed on a computer screen administered via an online survey platform (Qualtrics, https://www.qualtrics.com/).

### Eligibility criteria {10}

The eligibility criteria for participants are:

1. Participants must be full-time university students.
2. Participants should be able to read English, as the entire experiment will be conducted in English.
3. Participants must be aged 18–35 years old.
4. To ensure a minimum level of computer literacy, participants must use the computer on most days of the week.
5. To ensure that prior experience with the GRE would not confound the study results, participants must have NOT taken, or be preparing for, the GRE.
6. To avoid issues with eye-tracking data collection, participants should not wear glasses or have any eye impairment (such as cranial nerve III palsy). For those who wear contact lenses, they should not be colored and should not exceed 300 degrees.
7. Participants should not have any self-reported neurological or psychiatric disorders.

Participants who fulfill all eligibility criteria will be included in the study, while participants who do not fulfill the eligibility criteria will be excluded from the study.

Additionally, to participate in the study,

1. Participants should withhold alcohol 24 h before participation. Otherwise, they will be excluded from the study.
2. Participants should not wear makeup around the eyes, like mascara or eyeliner. If they do, they will be asked to wash it out before the experiment.

### Who will take informed consent? {26a}

Before the experiment starts, the participant will be given an information sheet and a consent form by the experimenter. The information sheet will explain the study’s aim, procedures, potential risks and benefits, compensation, and contact information of the study investigators. The experimenter will answer any questions that the participant may have before asking for consent. If the participant meets the inclusion criteria and agrees to participate, they will be asked to sign the consent form, which the experimenter will countersign. The participant will receive the information sheet and a copy of the consent form. The other copy of the consent form will be retained by the research team. All participants will be verbally informed that they can withdraw from the study at any time without giving any reason and without having any negative consequences to their academic studies.

### Additional consent provisions for collection and use of participant data and biological specimens {26b}

Not applicable. No data will be collected for ancillary studies.

## Interventions

### Intervention description {11a}

In the intervention group, the computer screen will be set up in a split-screen format. On the left side of the screen, the participant will receive instructions on the writing prompt, writing requirements, time requirements, grading feedback, and the grading rubric. The instructions will also highlight to the participant that they can use ChatGPT in any way they like to assist their writing, and there is no penalty in their writing score for how ChatGPT is used. The right side of the screen will display a blank ChatGPT interface where the participant can prompt questions and receive answers.

### Explanation for the choice of comparators {6b}

In the control group, as in the intervention group, the computer screen will be set up in a split-screen format. On the left side of the screen, the participant will receive the same instructions on the writing prompt, writing requirements, time requirements, grading feedback, and the grading rubric. Additionally, the instructions will highlight to the participant that they can use a text editor in any way they like to assist their writing. On the right side, instead of ChatGPT, a basic text editor interface will be displayed. In summary, this comparator will keep the split-screen format consistent between the two groups and ensure that participants in the control group will complete the writing task with minimal support.

### Criteria for discontinuing or modifying allocated interventions {11b}

This study is of minimal risk, and we do not anticipate needing to discontinue or modify the allocated interventions during the experiment. Participants can withdraw from the study at any time.

### Strategies to improve adherence to interventions {11c}

Adherence to the interventions will be high because the procedures are straightforward and will be clearly explained in the step-by-step instructions on the computer screen. The participant will be alone in a noise-canceling room during the entire experiment. The participant can reach out to the experimenter through an intercom if they need any clarification.

### Relevant concomitant care permitted or prohibited during the trial {11d}

Not applicable. This is not a clinical study.

### Provisions for post-trial care {30}

Not applicable. This is a minimal-risk study.

### Outcomes {12}

The study has two primary outcomes. First, we will measure participants’ writing performance scores on the analytical writing task. The task is adapted from the Analytical Writing section in the GRE, a worldwide standardized computer-based exam developed by the Educational Testing Service (ETS) [27]. Participants’ writing performance will be scored using the GRE 0–6 rubric and by an automatic essay-scoring platform called *ScoreItNow!*, which is powered by ETS’s e-rater engine [32, 33]. We chose to adapt from the GRE writing materials for two reasons. First, their writing task and grading rubrics were established writing materials designed to measure critical thinking and analytical writing skills and have been used in research as practice materials for writing (e.g. [34]). Second, OpenAI’s technical report shows that ChatGPT (GPT-4) can score 4 out of 6 (~ 54th percentile) on the GRE analytical writing task [31]. This gives us a benchmark for assessing the potential increase in writing performance when individuals collaborate with generative AI.

Second, we will measure participants’ cognitive effort during the writing process. Participants’ cognitive effort will be measured using a psychophysiological proxy—i.e., changes in pupil size [35, 36]. Pupil diameter and gaze data will be collected using the Tobii Pro Fusion eye tracker at a sampling rate of 120 Hz. During the preparation stage of the study, the room light will be adjusted so that the illuminance at the participants’ eyes is at a constant value of 320 LUX. Baseline pupil diameters will be recorded during a resting task in the experiment preparation stage that asks the participant to stare at a cross that will appear for 10 s each on the left, center, and right sections of the computer screen. Pupil diameters and gaze data will be recorded throughout the writing process.

**Fig. 2:**
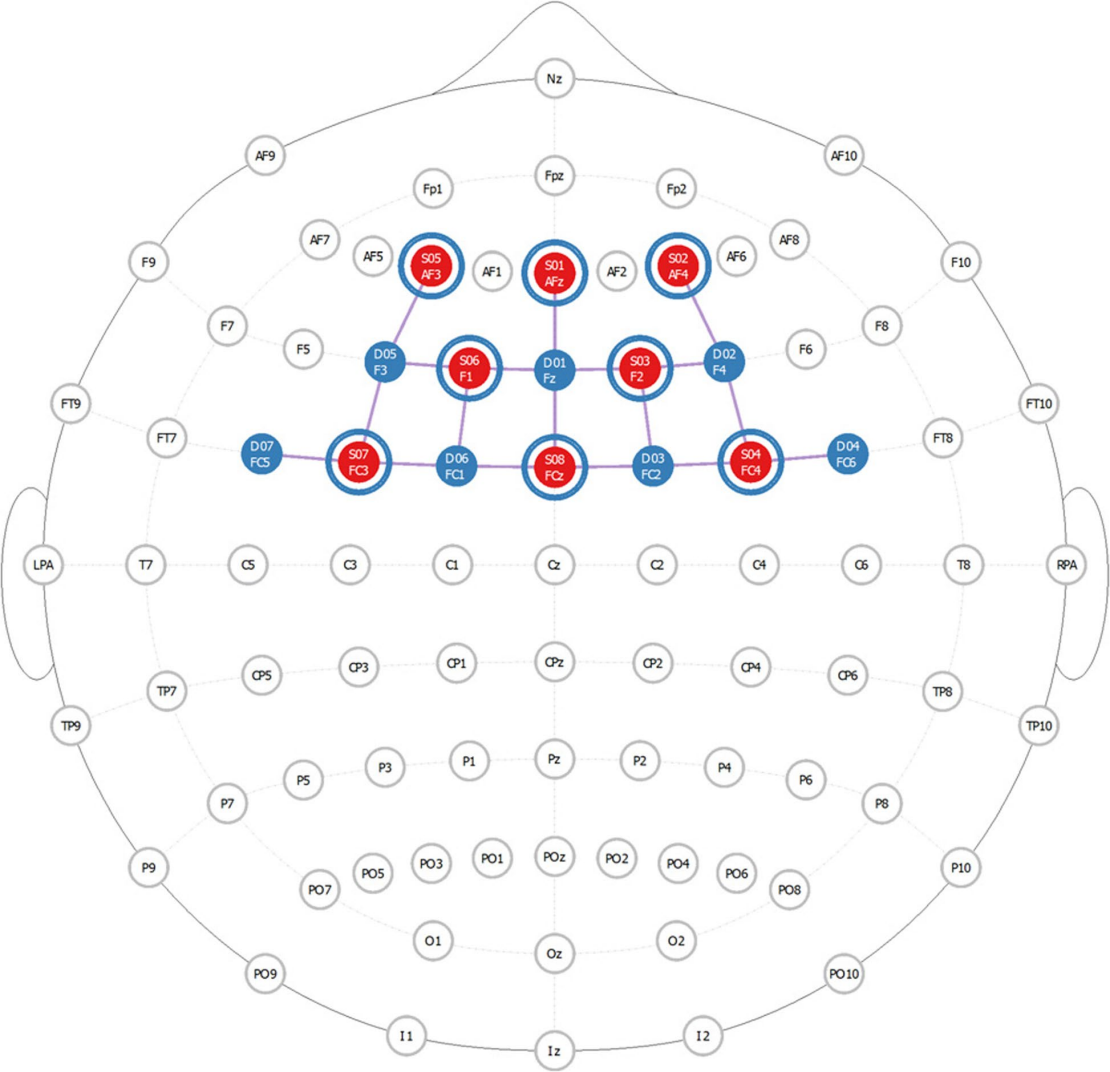
Design of the fNIRS montage

The study has several secondary outcomes. First, to identify the neural substrates of cognitive effort during the writing process, we developed an additional psychophysiological proxy, changes in the cortical hemodynamic activity in the frontal lobe of the brain. Specifically, we will examine hemodynamic changes in oxyhemoglobin (HbO). Brain activity will be recorded throughout the writing process using the NIRSport 2 fNIRS device and the Aurora software with a predefined montage (Fig. 2). The montage consists of eight sources, eight detectors, and eight short-distance detectors. The 18 long-distance channels (source-detector distance of 30 mm) and eight short-distance channels (source-detector distance of 8 mm) are located over the prefrontal cortex (PFC) and supplementary motor area (SMA) (Fig. 2). The PFC is often involved in executive function (e.g., cognitive control, cognitive efforts, inhibition) [37, 38]. The SMA is associated with cognitive effort [39, 40]. The sampling rate of the fNIRS is 10.2 Hz. Available fNIRS cap sizes are 54 cm, 56 cm, and 58 cm. The cap size selected will always be rounded down to the nearest available size based on the participant’s head measurement. The cap is placed on the center of the participant’s head based on the Cz point from the 10–20 system.

Third, we will measure participants’ subjective perceptions of the writing task by self-reported survey measures in the post-survey (Table 1). We will measure participants’ subjective perceptions of the two primary outcomes—that is, their self-perceived writing performance and self-perceived cognitive effort. Self-perceived writing performance will be measured with a one-item scale using the same grading rubric described in the instructions for their writing task and used in the scoring tool. Self-perceived cognitive effort will be measured using a one-item scale adapted from the National Aeronautics and Space Administration-task load index (NASA-TLX) [41, 42]. We will also measure participants’ subjective perceptions of several mental health and learning-related outcomes, including stress, challenge, and self-efficacy in writing. Self-perceived stress will be measured using a one-item scale adapted from the Primary Appraisal Secondary Appraisal scale (PASA) [43, 44]. Self-perceived challenge will be measured using a one-item sub-scale adapted from the Primary Appraisal Secondary Appraisal scale (PASA) [43, 44]. Self-efficacy in writing will be measured using a 16-item scale that measures three dimensions of writing self-efficacy: ideation, convention, and self-regulation [45]. Furthermore, we will measure participants’ situational interest in analytical writing using a four-item Likert scale adapted from the situational interest scale [46]. Additionally, we will measure participants’ behavioral intention to use ChatGPT in the future for essay writing tasks [47].

**Table 1:** Scales in the post-survey

Construct	Items	Response
Self-perceived writing performance	Using the same grading rubric from before, what score do you think your essay should get (0 being the lowest and 6 being the highest)?	0 to 6 scale
Self-perceived cognitive effort	On a scale of 1 to 7, rate how hard you had to work to accomplish your level of performance	1 to 7 scale
Self-perceived stress	On a scale of 1 to 7, how much would you agree or disagree with the following statement on perceived stress: The analytical writing assignment was stressful to me	1 to 7 Likert scale, 1 being “strongly disagree” and 7 being “strongly agree”
Self-perceived challenge	On a scale of 1 to 7, how much would you agree or disagree with the following statement on perceived challenge: I find the analytical writing assignment a challenge	1 to 7 Likert scale, 1 being “strongly disagree” and 7 being “strongly agree”
Self-efficacy in writing—ideation	On a scale of 1 to 7, how much would you agree or disagree with the following statements on coming up with ideas during your writing? 1. I could think of many ideas for my writing 2. I could put my ideas into writing 3. I could think of many words to describe my ideas 4. I could think of a lot of original ideas 5. I knew exactly where to place my ideas in my writing	1 to 7 Likert scale, 1 being “strongly disagree” and 7 being “strongly agree”
Self-efficacy in writing—convention	On a scale of 1 to 7, how much would you agree or disagree with the following statements on writing the essay properly? 1. I could spell my words correctly 2. I could write complete sentences 3. I could punctuate my sentences correctly 4. I could write grammatically correct sentences 5. I could begin my paragraphs in the right spots	1 to 7 Likert scale, 1 being “strongly disagree” and 7 being “strongly agree”
Self-efficacy in writing—self-regulation	On a scale of 1 to 7, how much would you agree or disagree with the following statements on regulating yourself during writing? 1. I could focus on my writing for the whole time 2. I could avoid distractions while I wrote 3. I could start the essay quickly 4. I could control my frustration when I wrote 5. I could think of my writing goals before I wrote 6. I could keep writing even when it was difficult	1 to 7 Likert scale, 1 being “strongly disagree” and 7 being “strongly agree”
Situational interest in analytical writing	On a scale of 1 to 7, how much would you agree or disagree with the following statements on your interest in the analytical writing assignment that you just completed? 1. The analytical writing assignment was interesting 2. Working on the essay was fun 3. I enjoyed writing the essay 4. The analytical writing assignment was enjoyable	1 to 7 Likert scale, 1 being “strongly disagree” and 7 being “strongly agree”
Behavioral intention to use ChatGPT in the future for essay writing tasks	On a scale of 1 to 7, how much would you agree or disagree with the following statements on using ChatGPT in essay writing assignments? 1. If I have access to ChatGPT, I would use it for essay writing tasks 2. I plan to use ChatGPT in the future if I have an essay writing task	1 to 7 Likert scale, 1 being “strongly disagree” and 7 being “strongly agree”

### Participant timeline {13}

**Fig. 3:**
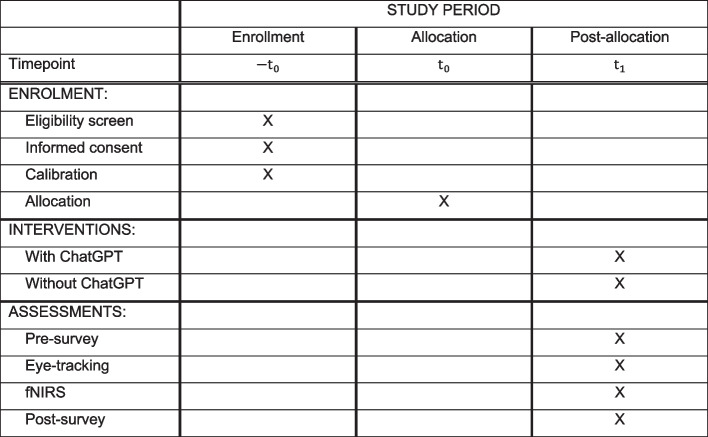
Schedule of enrollment, interventions, and assessments of the study

The time schedule is provided via the schematic diagram below (Fig. 3). The entire experiment will last for approximately 1–1.5 h for each participant.

### Sample size {14}

To estimate the required sample size, we conducted a simulation analysis on the intervention effect on writing performance using ordinary least squares (OLS) regression. Recent empirical evidence suggests that the effect size of generative AI on writing tasks ranges around Cohen’s *d* = 0.4–0.5, such as [1, 48]. In our simulation analysis, the simulated data assumes normally distributed data, equal and standardized standard deviations between the two conditions, and an anticipated effect size of Cohen’s *d* = 0.45. In the end, our analysis indicated that recruiting a minimum of 160 participants would be necessary to achieve a statistical power greater than 0.8 under an alpha level of 0.05. The simulation was implemented in R, and the corresponding code is available at the Open Science Framework (OSF) via https://osf.io/9jgme/.

We opt to base our sample size estimation on writing performance, but not on the other primary outcome, cognitive effort, for two reasons. First, the effect of generative AI on performance outcomes has been studied [1, 48], but we did not find prior evidence on the effect size of generative AI on cognitive effort using physiological measures. Second, our physiological measure of cognitive effort may likely be powered once the sample size satisfies our behavioral measure of writing performance. Pupillometry studies on cognitive efforts, such as the N-back test, typically recruit 20–50 participants in short, repeated, within-subject trials (e.g., [49]). These studies provide a general estimation of participants needed. Although our study design (i.e., a between-subject RCT) differs from common pupillometry studies, cognitive effort is still a repeated outcome measure using time series pupil data throughout the entire writing process. Repeated outcome measures generally can enhance statistical power by taking into account within-subject variability [50].

### Recruitment {15}

The recruitment will follow a convenience sampling strategy. To aim for a student population with diverse academic backgrounds, participants will be recruited broadly through social media platforms, email lists, and flyers at the research university where the experiment will be conducted. Given that the experiment will start during the summer, the research team can recruit summer school students as participants. Thus, the study sample will not be limited to the students presently at the university. The recruitment materials include a brief description of the study, the eligibility criteria for participation, and the compensation for participation. Individuals who are interested in participation can sign up on a calendar by selecting available time slots provided by the experimenters. Participants will receive 30 euros in compensation upon completion of the experiment. Participants who withdraw in the middle of the experiment will receive partial compensation, prorated based on the amount of time they spend in the experiment.

## Assignment of interventions: allocation

### Sequence generation {16a}

The sequence will be generated using computer-generated random numbers to assign participants in a 1:1 ratio to the intervention group or the control group. The randomization process will be independent of the recruitment and implementation process. Only participants who fulfill the eligibility criteria and give consent to participate in the study will be allocated to the randomized sequence.

### Concealment mechanism {16b}

Not applicable. The randomization procedures are covered in Sects. 16a, 16c, and 17a.

### Implementation {16c}

Randomization was generated in advance of the entire experiment using an R script that allocated participant IDs into either the intervention group or the control group. The randomization algorithm is independent of the researchers who will recruit participants and implement the protocol.

## Assignment of interventions: blinding

### Who will be blinded {17a}

Participants will be blinded to the randomization process but not to the intervention itself. Specifically, participants are not informed that the study involves a randomized controlled trial or that there are different experimental conditions. As such, they are unaware of whether they have been assigned to a control or intervention group. However, blinding to the intervention is not feasible in this context: participants in the intervention group are explicitly instructed to use ChatGPT during the writing task. Experimenters will not be blinded because they need to set the computer screen to the appropriate format depending on whether the participant is assigned to the intervention group or the control group. The analyst will be blinded, and the assignment condition will be masked from the analyst to minimize potential biases from statistical analysis.

### Procedure for unblinding if needed {17b}

Unblinding participants to the randomization process is not permissible because it may introduce social desirability bias. That is, if participants are aware that they are assigned to the intervention group, they may act differently to align with expectations of the experimenter. The participant’s data will be excluded if the assigned condition is accidentally revealed and will not be counted in the randomized sequence.

## Data collection and management

### Plans for assessment and collection of outcomes {18a}

Data will be collected from multiple sources: pre- and post-survey responses as well as the final essay via the Qualtrics platform, interaction logs with ChatGPT for the intervention group, writing data in the text editor for the control group, gaze and pupil-related data from the eye tracker, and hemodynamic activity data from the fNIRS. In particular, the surveys will capture participants’ background characteristics that may contribute to heterogeneity effects (Table 2). The pre-survey will include measures such as participants’ initial interest in analytical writing and their motivation to achieve a high score on the writing task. The post-survey will include participants’ frequency of ChatGPT use, English ability, their native language, writing ability, critical thinking ability, and demographic details, including their age, gender, race, academic year, and academic major. Background characteristics are mainly collected in the post-survey, unless necessary to be in the pre-survey, to avoid potential signaling effects that could influence participants’ writing behavior during the writing task. The data retrieved from all sources will be anonymous. Data downloaded will be stored on an encrypted and secure server. The data will be deleted 5 years after the study has been completed.

**Table 2:** Background information in the pre- and post-surveys

Survey	Construct	Items and response
Pre-survey	Initial interest in analytical writing	On a scale of 1 to 7, how much do you agree or disagree with the following statements? (1 being “strongly disagree” and 7 being “strongly agree”) 1. I find analytical writing enjoyable 2. I enjoy writing an analytical essay 3. I like learning new skills about analytical writing
	Motivation to achieve a high score in the writing task	On a scale of 1 to 5, how motivated are you to achieve a high score in your essay? (1 being “not at all motivated” and 5 being “extremely motivated”)
Post-survey	Frequency of ChatGPT use	In the past month, how often have you used ChatGPT for writing tasks? Consider all forms of writing tasks, including but not limited to analytical essays, academic papers, reports, reading reflections, and discussion posts. Note that there are no requirements on how you used ChatGPT for these tasks • Never (not used it or not heard of it) • Occasionally (1–3 times a week) • Everyday or almost everyday (> 3 times a week)
	English ability	What is your level of English proficiency? • My English level is basic (I can describe simple terms related to areas of immediate need) • My English level is intermediate (I can produce clear, detailed text on a wide range of subjects) • My English level is proficient (I can produce text spontaneously and precisely) • I am a native speaker of English
	Native language	What is your native language? (This question will be skipped if the participant answered “I am a native speaker of English” in the English ability question.)
	Writing ability	In the past month, how often have you written essays? • I rarely write essays • I write occasionally for academic purposes (e.g., class assignments) • I regularly write for academic or work-related tasks (e.g., reports, academic papers) • I write daily as a significant part of my job or academic work
	Critical thinking ability	How much are you trained in critical thinking? • I have never received any formal training in critical thinking • I have taken workshops or courses that emphasizes critical thinking • I have a degree in a field that emphasizes critical thinking • I have extensive formal or professional experience in critical thinking
	Age	What is your age?
	Gender	What is your gender? • Man • Woman • Non-binary • Prefer not to say
	Race/ethnicity	What is your race or ethnicity (select all that apply)? • Asian • Black/African • Hispanic/Latino • White/Caucasian • Other, please specify:
	Academic year	What academic year are you in? • Undergraduate, first-year of bachelor’s degree • Undergraduate, second-year of bachelor’s degree • Undergraduate, third-year of bachelor’s degree • Undergraduate, fourth-year of bachelor’s degree • Undergraduate, fifth-year or above of bachelor’s degree • Graduate, master’s degree • Graduate, doctoral degree • Law degree • Medical degree • Other, please specify: • Not applicable
	Academic major	What is your major? • My major is: • I’m undecided

### Plans to promote participant retention and complete follow-up {18b}

For each participant, the study will take approximately 1.5 h. The participant may withdraw from the study at any time. Their compensation will be rounded based on the amount of time they spend in the experiment. There is no follow-up study.

### Data management {19}

All data collection during the experiment will be anonymous. The experimental data collection process will be separated from the collection process for the personally identifiable data required for scheduling and consent purposes. Pseudonymized IDs will be used to join all data sources. Survey data will be collected on the Qualtrics platform. Except for Qualtrics, third parties will not have access to this data. Interaction log with ChatGPT will be collected on the ChatGPT platform under the study team’s account, exported, and removed from the platform after each participant completes their session. All other data (e.g., eye-tracking data, fNIRS data, writing data in the text editor) will be locally collected on the computers in the experiment room. All data will be uploaded to the university-owned, encrypted cloud storage service. Only the study team will have access to the data.

### Confidentiality {27}

Data collected on the survey, ChatGPT, and the local computer will be joined using pseudonymized IDs for data analysis. Participants’ personal information (i.e., name, contact information, and experiment time slot) will be collected separately only for contacting and scheduling purposes, and in case the participant would like to withdraw their data from the study. The principal investigator and research staff who conduct the experiment will have access to this information and have been trained before the study to ensure that they understand the rules for confidentiality and data protection. No other researchers on the team will have access to the data on personal information. No data will be captured on paper/physical media other than the signed consent form and the compensation confirmation form. The two forms will be stored in a locked cabin in the experiment room.

### Plans for collection, laboratory evaluation, and storage of biological specimens for genetic or molecular analysis in this trial/future use {33}

Not applicable. No biological specimens will be collected for the study.

## Statistical methods

### Statistical methods for primary and secondary outcomes {20a}

Here, the intervention effect on the two primary outcomes will be estimated by an intent-to-treat analysis. Our first primary outcome, writing performance, will be treated as a continuous variable. Our second primary outcome, cognitive effort as measured by mean changes in pupil size, will also be treated as a continuous variable. We will estimate the intervention effect on each outcome separately using ordinary least squares (OLS) regression with heteroskedasticity-robust standard errors. To control for type I error inflation due to multiple testing, we will apply a Bonferroni correction. Accordingly, the adjusted significance threshold is set at *α* = 0.05/2 = 0.025. The outcome of cognitive effort is a psychophysiological measure recorded throughout the entire writing process. Standard pre-processing steps will be taken before the statistical analysis [51, 52], such as correcting for screen angle distortion, removing invalid data that do not reflect the actual pupil size (pupil size below 1.5 mm or over 9 mm [53], often due to blinking), smoothing to remove small oscillatory and noise activity, interpolating blink related invalid data, downsampling, and correcting pupil size changes based on the baseline pupil size that will be collected during a 30-s relaxation task at the beginning of the experiment. Participant-level data will be excluded if the data do not meet basic quality standards, such as having a high proportion of invalid data.

For the secondary outcomes, all survey scale measures will be treated similarly to the writing performance outcome. They will be viewed as continuous variables and analyzed using OLS regression with heteroskedasticity-robust standard errors. Cognitive effort, as measured by changes in the cortical hemodynamics, will be treated similarly to pupil size changes. Through a series of pre-processing steps, we will compute channel-wise hemodynamic changes in HbO using the Satori software. The raw data of two wavelengths (760 nm and 850 nm) will be trimmed so that only the fNIRS data collected during the writing task is analyzed, and bad channels will be rejected using a coefficient of variation (CV) [54]. Then, the data will be further pre-processed using a standard pipeline, including data conversion using modified Beer-Lambert law (MBLL) [55], spike removal using a robust spike detection method, motion correction using the Temporal Derivative Distribution Repair (TDDR) [56], physiological noise removal using the highest correlated channel data from the eight short channel data, temporal filtering, and normalization through the z-normalization step. After pre-processing, we will use a generalized linear mixed model to estimate the intervention effect to account for participant-level random effects.

For the above statistical modeling, we will first run the analyses without adjusting for covariates because randomization, on average, eliminates confounding. Subsequently, we will run the analyses with adjusted covariates, including participants’ self-reported skill levels and motivation, because power can often be improved with covariate adjustments, and such adjustments can improve residual confounding. For skill levels, we will include three variables based on the three aspects of self-reported skill level: writing ability, critical thinking ability, and English language ability. For motivation, we will include one variable based on participants’ self-reported motivation to achieve a high-performance score in the analytical writing task. The four variables will be viewed as continuous variables and all added to the model. Should there be variations in other baseline measures, such as gender and race, between the intervention group and the control group, we will further adjust our model to control for these potential confounding sources.

### Interim analyses {21b}

We do not plan to conduct any interim analyses.

### Methods for additional analyses (e.g., subgroup analyses) {20b}

We will estimate complier average causal effects (CACE) on the primary outcomes. We will also conduct subgroup analyses to examine heterogeneous intervention effects, provided that sufficient sample sizes can be recruited in each group. The variables of interest are prior skill levels in writing ability, critical thinking ability, and English language ability, as well as motivation. Each of these variables will be examined independently. Additionally, we will take advantage of the pupil size data collected throughout the writing task to explore the intervention effect on cognitive effort across various self-allocated sub-tasks, such as writing the essay, reading the essay, and prompting ChatGPT.

### Methods in analysis to handle protocol non-adherence and any statistical methods to handle missing data {20c}

During the lab experiment, we will monitor and control for missingness in the survey data due to non-adherence under 20%. If the missingness is less than 5%, we will conduct a complete case analysis, which removes participants with missing responses [57, 58]. If the missingness falls between 5 and 20%, we will apply multiple imputation using the rest of the survey data [59].

### Plans to give access to the full protocol, participant-level data, and statistical code {31c}

This document is the full protocol. Anyone interested in aggregated versions of the data and the statistical code may contact the corresponding author. The consent form and other materials can be accessed via OSF: https://osf.io/9jgme/.

## Oversight and monitoring

### Composition of the coordinating center and trial steering committee {5d}

The coordinating center will be based at the Heidelberg Institute for Global Health (HIGH). The day-to-day experiment coordination will be managed by the study team at the Core Facility for Neuroscience of Self-Regulation (CNSR). The principal investigator will provide oversight of the study. The data manager will be responsible for organizing data collection and ensuring the integrity and quality of the data. The study coordinator will oversee participant recruitment, study visits, and weekly feedback reports. There is no trial steering committee or stakeholder and public involvement group.

### Composition of the data monitoring committee, its role and reporting structure {21a}

The study will not include a data monitoring committee separate from the study team because there will be no interim data analyses. The study team is independent from the sponsor of the trial and competing interests.

### Adverse event reporting and harms {22}

This trial is a lab experiment that asks participants to complete a writing task. It is very unlikely to cause adverse events.

### Frequency and plans for auditing trial conduct {23}

Not applicable. This study is a small-scale lab experiment that does not require external auditing.

### Plans for communicating important protocol amendments to relevant parties (e.g., trial participants, ethical committees) {25}

In the event of substantial amendment, this will be reported to the Ethics Committee at Heidelberg Medical Faculty. Non-significant amendments will be documented and updated in the online trial registries. Additional documents will be uploaded to the OSF.

### Dissemination plans {31a}

The results of this study will be disseminated through presentations at international conferences and publications in peer-reviewed journals.

## Discussion

Since the public release of ChatGPT in 2022, there have been heated discussions on the societal implications of generative AI. Concerns and promises have both been raised about its potential effect on human cognition when such tools are widely integrated into daily tasks [6, 15]. In this study, we propose to evaluate the effects of generative AI use on human cognition and task performance, in the context of a hypothetical analytical writing assignment undertaken by college students.

The main innovation of our study is using multi-modal data to evaluate the effects of generative AI. We will collect psychophysiological data throughout the writing process using state-of-the-art neuroscience technologies. Specifically, we will use the Tobii Pro Fusion eye tracker to capture pupil size changes and gaze patterns. We will use the NirSport2 fNIRS system to measure brain activity. These data will then be combined and analyzed with behavioral data and self-reported attitudinal data collected in the pre- and post-surveys. The multi-modality of the data provides a few advantages. First, collecting data from different modalities will give us a more comprehensive understanding of the effects of generative AI. For example, combining psychophysiological measures with self-reported measures can provide insights into both the internal cognitive processes and observable behaviors of participants. Second, multi-modal data will allow us to validate findings from different data sources. Third, the real-time measures captured in this study reflect dynamic changes as tasks are performed. These data will provide deeper insights into how cognitive processes evolve during different phases of a task.

Our study design ensures a high internal validity due to the controlled lab setting. However, this approach has limitations in generalizability. The recruitment process relies on a convenience sampling strategy, as the experiment requires equipment located at the university’s research lab. As a result, participants may represent a WEIRD (Western, Educated, Industrialized, Rich, Democratic) population and may not represent the impact of generative AI use in a broader, more diverse population. Moreover, participants in the experiment may not behave as they would in real-world settings. In our study, we will carefully control the motivational context for the writing task. Specifically, we will measure participants’ general motivation to achieve a high score before they start working on the writing task and will account for this variation in our regression models. We will also control for external incentives by framing the experiment setting as a hypothetical writing class and by informing participants that they will receive their performance scores, the class average, and the instructor’s feedback after completing the task. This design aims to reflect real-life motivational settings for completing homework assignments. Unlike in other experimental research evaluating the effects of generative AI (e.g., [1, 4]), we opt not to incentivize better performance monetarily, as this is not suitable for our study context.

## Data Availability

The final trial data are deidentified and will be stored on a university-owned, encrypted cloud storage service. The study investigators own and have complete control over the research data. Open Access funding enabled and organized by Projekt DEAL.

## Abbreviations

AI: Artificial intelligence
SAP: Standard assessment paradigm
RCT: Randomized controlled trial
fNIRS: Functional near-infrared spectroscopy
GRE: Graduate Record Examination
ETS: Educational Testing Service
HbO: Oxyhemoglobin
PFC: Prefrontal cortex
SMA: Supplementary motor area
NASA-TLX: National Aeronautics and Space Administration-task load index
PASA: Primary Appraisal Secondary Appraisal
OLS: Ordinary least squares
OSF: Open Science Framework
CV: Coefficient of variation
MBLL: Modified Beer-Lambert law
TDDR: Temporal Derivative Distribution Repair
CACE: Complier average causal effects
HIGH: Heidelberg Institute for Global Health
CNSR: Core Facility for Neuroscience of Self-Regulation
WEIRD: Western, Educated, Industrialized, Rich, Democratic

## Declarations

### Trial status

This trial is currently recruiting participants. Recruitment for the trial and all data collection will be completed by the end of Feb 2025.
